# Cases and Observations Illustrative of Renal Disease, Accompanied with the Secretion of Albuminous Urine

**Published:** 1836-07-01

**Authors:** 


### Cases and Observations illustrative of Renal Disease, accompanied with the Secretion of Albuminous Urine. By *Dr. Bright.*

[Guy's Hospital Reports, No. 2.]

The importance of the subject, and the ability of the writer, demand a separate article for this report; and, on that account, we have placed it in our Review, instead of Periscopic department. It might be difficult to determine whether the disease in question has become more frequent of late years, or only been better observed and oftener detected. For our own parts, we think that the disease called "mottling," "white degeneration," "contraction," or "granulation," *is* of more common occurrence than formerly, and *is also* better understood, both as to symptoms and pathology. Dr. Bright, who has paid especial attention to this dangerous malady for ten or a dozen years past, is of opinion that 500 people fall victims to it every year in London alone. He thinks there is great reason to believe that the seeds of the disease are often early sown, and that intervals of apparent health produce a false feeling of security.

Hæmaturia is often a precursor of renal disease—and so is scarlatina. Intemperance, however, is its most common source—and exposure to cold the most usual exciting cause. Intemperance in youth generally lays so firm a foundation for the malady, as to bid defiance to remedies afterwards. The history of this disease and its symptoms are nearly as follow:—

"A child, or an adult, is affected with scarlatina, or some other acute disease; or has indulged in the intemperate use of ardent spirits for a series of months or years; he is exposed to some casual cause or habitual source of suppressed perspiration: he finds the secretion of his urine greatly increased, or he discovers that it is tinged with blood; or, without having made any such observation, he awakes in the morning with his face swollen, or his ankles puffy, or his hands œdematous. If he happen, in this condition, to fall under the care of a practitioner who suspects the nature of his disease, it is found, that already his urine contains a notable quantity of albumen: his pulse is full and hard, his skin dry, he has often headache, and sometimes a sense of weight or pain across the loins. Under treatment more or less active, or sometimes without any treatment, the more obvious and distressing of these symptoms disappear; the swelling, whether casual or constant, is no longer observed; the urine ceases to evince any admixture of red particles; and, according to the degree of importance which has been attached to these symptoms, they are gradually lost sight of, or are absolutely forgotten. Nevertheless, from time to time, the countenance becomes bloated; the skin is dry; headaches occur with unusual frequency; or the calls to micturition disturb the night's repose. After a time, the healthy colour of the countenance fades; a sense of weakness or pain in the loins increases; headaches, often accompanied by vomiting, add greatly to the general want of comfort; and a sense of lassitude, of weariness, and of depression, gradually steal over the bodily and mental frame. Again the assistance of medicine is sought. If the nature of the disease is suspected, the urine is carefully tested; and found, in almost every trial, to contain albumen, while the quantity of urea is gradually diminishing. If, in the attempt to give relief to the oppression of the system, blood is drawn, it is often buffed, or the serum is milky and opaque; and nice analysis will frequently detect a great deficiency of albumen, and sometimes manifest indications of the presence of urea. If the disease is not suspected, the liver, the stomach, or the brain divide the care of the practitioner, sometimes drawing him away entirely from the more important seat of disease. The swelling increases and decreases; the mind grows cheerful, or is sad; the secretions of the kidney or the skin are augmented or diminished, sometimes in alternate ratio, sometimes without apparent relation. Again the patient is restored to tolerable health; again he enters on his active duties: or he is, perhaps, less fortunate;—the swelling increases, the urine becomes scanty, the powers of life seem to yield, the lungs become œdematous, and, in a state of asphyxia or coma, he sinks into the grave; or a sudden effusion of serum into the glottis closes the passages of the air, and brings on a more sudden dissolution. Should he, however, have resumed the avocations of life, he is usually subject to constant recurrence of his symptoms; or again, almost dismissing the recollection of his ailment, he is suddenly seized with an acute attack of pericarditis, or with a still more acute attack of peritonitis, which, without any renewed warning, deprives him, in eight and forty hours, of his life. Should he escape this danger likewise, other perils await him; his headaches have been observed to become more frequent; his stomach more deranged; his vision indistinct; his hearing depraved: he is suddenly seized with a convulsive fit, and becomes blind. He struggles through the attack; but again and again it returns; and before a day or a week has elapsed, worne out by convulsions, or overwhelmed by coma, the painful history of his disease is closed." 341.

Here a question may be asked, is the said derangement of kidney necessarily connected with the peculiar condition of urine described? After ten years' observation, Dr. B. thinks he may safely answer in the affirmative—although anomalies will sometimes occur. The cases which our author adduces will be found to bear upon, and illustrate many points in the history which has been sketched in the foregoing extract. We shall endeavour to condense the more important features of several of these cases.

*Case* 1. A physician, aged 42, who had lived freely, but not intemperately, applied to Dr. B., in the year 1832, with marked symptoms attendant on albuminous urine—his flesh wasting and strength failing. His legs were œdematous. These symptoms had continued for two years, and he had been treated for diabetes. The urine was highly albuminous. The decoction of pylora umbellata was prescribed; and, after some days, two grains of salicine thrice a day were also ordered. He improved a little under this treatment, with regulated diet, and proceeded to North America, where he had formerly resided some years. In October, 1833, he presented himself to Dr. B. in apparent good health. He had been more than a year in Canada, where his health improved. In England, however, some of his former symptoms returned.

" His legs began to swell, so that he was obliged always to wear bandages: his hands also swelled occasionally: he had great inability to sleep at night: suffered from vertigo: his urine was coagulable by heat, though it emitted a urinous smell: appetite good: tongue clean: pulse from 80 to 90: skin freely perspirable. He kept his bowels regular, by pills. He remained in tolerable health; but was always excessively low spirited, and suffered much from headache: he had constantly a slight tendency to anasarca, and his urine was always albuminous.

In the latter part of the summer of 1834, he again resolved, at all hazards, to try the effect of revisiting America; and he seemed, to a certain degree, recruited by the voyage. But in the latter end of November, having exposed himself a good deal in shooting, he was attacked with hemiplegia; and in three weeks after, died apoplectic, on the 15th of December." 345.

In this case the precise disorder which occasioned the albuminous urine was not, of course, ascertained. Its existence was traced over a space of four years, during which he never could be said to have enjoyed perfect health, though with considerable intervals of amelioration.

*Case* 2. July 1835, a medical gentleman from the country, aged 33, consulted Dr. B. His countenance was pallid. He said he enjoyed good health till the age of twenty, when he became affected with dyspepsia, which had gradually increased. In 1826, he had the ulcerated sore throat of scarlatina, without rash. His urine was then as deep coloured as coffee. He believed his urine had been albuminous since that period.

" Six years ago he began to suffer from occasional pain in the back and region of the kidneys, rather more on the left than the right side; and he had wandering pains about the heart, which were considered to be neuralgic. The latter symptoms subsided, but the dull pain in the back continued; and he had since been subject to puffy œdematous swelling of the face, legs, and ankles. Four years ago he had an attack of catarrhal inflammation of the chest, which appeared to have rendered him very susceptible of atmospheric changes; and about a year and a half ago he wasted considerably. He had suffered, for the last fifteen years, from severe attacks of sick headache, with vomiting, which continued for several hours; and this had increased during the last few months. The fluid which was ejected from his stomach was excessively acid, and sometimes tinged with bile: the bowels were habitually costive. I understood that he has been temperate as regards drink, but irregular in his meals. I examined his urine carefully, found it of a light straw colour, very acid, and throwing down numerous flocculi of albumen by the application of a moderate degree of heat. He evidently saw that I considered his case one of a most serious character; and he consented to do any thing I would direct, consistently with his continuing to practise his profession. I enjoined upon him the greatest care in avoiding exposure to cold and chills; told him to clothe himself completely in flannel; to keep strictly to a milk diet; and to regulate his bowels with scammony or some simple purgative; and I prescribed powders of the uva ursi, soda, and compound ipecacuanha powder, to be taken thrice a day." 346.

Dr. B. saw him on the 1st October, of the same year, considerably improved; but the urine was still coagulable. He ascribed his improvement chiefly to a rigid adherence to milk diet, and aperients. Yet in eight weeks from this time he died.

" I learned, that about six weeks before his death he had suffered most severely from headache and vomiting; which was now accompanied by dimness of sight, or a kind of mistiness, as he described it; and a whizzing in his ears, which was not more on one side than on the other. During the last month, while he was still going about, he complained of an inability to direct his arms and legs, which affected the left rather than the right side. For the last fortnight he kept his bed; and during that time he had frequent twitchings of different muscles; and generally at night, when he fell asleep, was attacked with a convulsion, which almost raised him from his bed. On the fifth day before his death he had three attacks of epilepsy, and one or two on the following days: during the last two days of his life he lay in a comatose state." 347.

The body was carefully examined by Mr. Hilton. There was a diseased condition of the mucous membrane of the duodenum, advancing towards ulceration. "The kidneys presented minutely mottled degeneration in their secreting structure, as depicted by Dr. Bright in his medical reports; but neither of them was softened, nor much increased in size. The ureters and bladder were healthy." The heart was large and flabby. The walls of the left ventricle were hypertrophied, but flaccid—the fibres pale, and easily lacerable. There were several traces of disease in the head.

"On raising the dura mater carefully, there was a layer of coagulated blood recently effused, about the side of a half-crown, resting between the two layers of the arachnoid membrane; not firmly adherent to either, but more so to the arachnoid of the dura mater, corresponding to the external cranial depression where that membrane was flaccid. Arachnoid opaque and thickened (looking like wet, thin, white leather), from chronic inflammation over nearly the whole of the vertex on each side; rather more on the left; with some little serous effusion under the arachnoid. A small deposit of bone upon, or rather growth of bone from, the arachnoid, on the pia mater, somewhat larger than a pea, was observed under the loose portion of the dura mater, presenting a superior, nodulated, uneven surface. There were also several cartilaginous spots, but none larger than the section of a pea, all on the left hemisphere of the brain. A portion of the falx major, near its junction with the tentorium, was ossified to about the extent of a sixpence, with irregular edges. The pia mater was not adherent, except over the superior part of the left anterior cerebral lobe, where it was slightly so, and some patches of cineritious structure came off with it: and at this point, the convolutions were not so prominent as at the corresponding part on the right side." 350.

The following commentary of Dr. Bright on this melancholy case we shall give in his own words, as they cannot be abridged.

"The whole history of this case is most interesting; shewing, as it appears to do, the connexion of this disease, in the first place, with the scarlatina; presenting traces of it through its lengthened progress, attended by occasional indications in the obvious character of the urine, in the slight and often evanescent œdema of various parts, in the tendency to emaciation, in the returns of vomiting and severe headache, and, at last, in the more decided cerebral symptoms; which, after a succession of convulsive seizures, terminated in coma, when probably eight years, at least, had elapsed from the first commencement of the disease." 351.

CASE 3.—*Dec. 25th*, 1835. Dr. B. was hastily summoned to meet Dr. Prout, in the case of Mr. W. aged 17 years. He was found in bed, with his eyes half closed—his left arm paralytic, as also the left leg—countenance pale—tongue furred and dry—surface warm—complaining of pain in the head—acute sense of hearing. His urine was pale, acid, and very coagulable by heat. He now had a convulsive fit, during which the paralyzed members were violently agitated, while he cried out with pain. He was quite conscious. The paroxysm lasted only a few minutes, when he lapsed into his former state of tranquillity. When 12 years of age, he had an attack of hæmaturia, which had recurred several times, with copious flow of urine, and desire to pass it often. A small calculus had once passed. Dr. Prout saw him 18 months ago, when the urine was coagulable, in which condition it has remained. Three weeks ago he was much exposed to cold at a funeral, since which he has got worse.

"He was seen by Mr. Odling about ten days ago, who found that he had suffered more headache than usual: bowels constipated: tongue brown, and loaded. He was ordered leeches to the temples, took purgatives, and got so much better, that on Saturday the 19th he was occupied in some business, which required great exertion both of mind and body, and exposed him to the cold; and he found himself so much confused, that he retired to bed; and when he was found there, about nine o'clock, he was affected with a most intense headache, vomiting, and a total loss of sight. Mr. Odling, seeing him in this state, got a large number of leeches; but whilst he was in the act of applying them to his temples, a severe fit came on, of an apoplectic character, in which the patient's countenance became dark and suffused, and his breathing stertorous. He was bled freely from the arm; and this was repeated three times during the night; but he experienced two more fits, like the first. In the morning, Dr. Prout saw him, and ordered cupping from the loins; and whilst this was being done, a fit occurred. On the Tuesday, fits, assuming more the character of epilepsy, occurred; in which the left side, which had remained weak after the first fits, was convulsed. On Wednesday, the fits returned; but it was evident that the mind was unimpaired during the convulsion. On that night they were frequent; and on the following day not less than twenty occurred. On Friday the 25th, the day on which I was called, they had recurred at intervals of about an hour, and were rapidly increasing. At eight o'clock in the evening, he became quite insensible; but he lived, with frequent convulsions, till two o'clock on Saturday morning; thus lingering nearly a week from the first appearance of the severe cerebral disease; during the last five days of which time, it was necessary to draw off his water every eight hours." 353.

No examination was permitted; but there can be little doubt of the nature of the disease. The following case was communicated by Mr. Weatherfield, an intelligent surgeon of this metropolis.

*Case* 4. Mr. J. G. consulted the narrator in the month of December, 1830, for dyspepsia, accompanied by pyrosis. He was again seen in Sept. 1833, when a total change had taken place in the symptoms.

"He had for some time lost the dyspepsia and pyrosis; his appetite was good; and, to use his own words, he could digest any thing: but complained of pain and throbbing in his head, intolerable thirst, huskiness in the throat, swelling of the legs towards night, and of the face in the morning: his pulse was now hard: tongue covered with a creamy coat: urine very abundant. He was advised to be bled; but he neither submitted to that, nor pursued any plan of treatment steadily. He continued much in the same state, till February 1834; when the pain in the head became less frequent, but more severe, returning generally once a week, attended with distressing vomiting: the œdema of the face and extremities somewhat increased: there was now, also, some fluid in the cavity of the abdomen. Soon after this, he took a long journey into the country, where he underwent great fatigue, and suffered from a more severe attack of head affection and vomiting than before; which induced him to return to town, and consult Dr. Babington, which he did on the 26th of March. After a very careful and patient investigation of the case, Dr. Babington, having tested the urine, and finding it to contain albumen, declared it his opinion that the kidney was the seat of the disease. He was ordered to take alterative mercurials and tonics; and to be cupped over the region of the liver, where he complained of pain, increased upon pressure. The following week he saw Dr. Babington again; when he complained of noise in the left ear, and slight numbness of the right hand and foot: the pulse at this time was feeble. Dr. Babington, however, ordered blood to be taken away, if any aggravation of symptoms should occur. On the morning of the 5th of April he was attacked with epilepsy; and a medical gentleman living near immediately bled him. The pulse rose wonderfully, and continued so strong as to induce us to repeat the bleeding the next day; which, together with purgatives, subdued the convulsions for a time. On the 11th of April, however, he was suddenly seized with another fit; which returned at longer or shorter intervals, until the following day, when he sunk.

*Sectio Cadaveris.* The membranes of the brain, particularly the pia mater and arachnoid, much thickened; more than two ounces of water in the ventricles; the brain being altogether firmer than usual: the septum lucidum thickened and tough: plexus choroides paler than usual.

The lungs were emphysematous; the air-cells much enlarged, and ruptured.

The heart rather larger than usual; the left ventricle somewhat thickened; and the semi-lunar valve slightly ossified: some water was found in the cavity of the chest.

All the abdominal viscera healthy, except the kidneys; which were much smaller than usual, of a grey and granulated structure." 355.

*Case* 5. Mr. P. aged 25, athletic, applied to Mr. Wheelwright on the 6th December, 1835, for dyspepsia, with dimness of sight. The symptoms being referred to bilious disorder, aperients, followed by bitter tonics, were administered. He got better, and returned to his employment, that of a plumber. He got cold on the top of a building, on the 21st January of the present year, and returned home ill, with a kind of convulsive fit. Mr. Wheelwright saw him at five o'clock in the morning, when he was totally blind—pulse quick and powerful—intense pain in the head. He was bled copiously, and a brisk purgative was given. The bleeding was repeated, and cupping was employed, as the headache continued. Dr. Babington saw him the following day, when considerable arterial action was still present. As he had lost 40 ounces of blood, it was determined to try the effects of mercury. In four or five days his vision was somewhat improved, and the headache relieved. Leeches and blisters were ordered. In four days more the mouth was affected. He was extremely drowsy, and this increased to coma, and ended fatally. The body was examined a few hours after death. About half an ounce of fluid was found in the ventricles of the brain, and some degree of ramollissement about the basis cerebri. The optic nerves were healthy, as were all the viscera of chest and abdomen, except the kidneys, the peritoneal and proper tunics of which were thickened and opake. When stripped off, the surface beneath was rough, and of a grey mottled appearance. The diseased structure was seen to extend throughout the substance of the organ on section. Both kidneys were equally affected. The urine had not been examined in this case, as there had been no tendency whatever to dropsy.

*Case* 6. This was a young woman, who had presented albuminous urine for five years. Her blood had been analysed and found to contain urea. Latterly her head became affected, and she had epileptic fits. Her complexion was pale, yet she seemed well nourished. She was walking about when seized with a fit which terminated her existence. On examination, the skull was found to be unusually thick, and there were one or two small exostoses on its inner surface. A copious effusion was found under the arachnoid, and the cineritious substance of the brain was injected. The lining membrane of the ventricles was thickened. The viscera presented nothing very particular. The kidneys were small, rather granular on their surface, and of a firm dense texture.

*Case* 7. A young woman was admitted under Dr. B. with profuse general anasarca and coagulable urine. She had been chlorotic for some years, with irregular menstruation. Fifteen months previously to admission her legs began to swell, after catching cold. The urine was now exceedingly coagulable, and her headaches were intense—often attended by vomiting.

" This young woman remained under my care for many months, varying almost every week in a remarkable degree, and subject to repeated relapses of anasarca, which she almost always ascribed to some slight exposure, generally to the draught of an open window. She was kept on the mildest diet; chiefly milk, and beef-tea and arrow-root. The remedies employed were principally diaphoretics; amongst which, the warm-bath, the hip-bath, and Dover's powder were the most important. From the beginning of May to the beginning of September, I confined her entirely to her bed; and frequently obtained a very perspirable state of skin, sometimes almost in excess. This treatment was often suspended and varied, owing to the severe attacks of vomiting. After a time, the catamenia began to return with tolerable regularity. The flow of urine was often very abundant, amounting to four pints in the twenty-four hours, and varying somewhat in the extent of its albuminous qualities, but never quite free from albumen: and at length, the anasarcous swellings being reported as entirely gone, she became very desirous of returning home. I however detained her several days, till her mother had provided her with a flannel dress, to wear constantly close to the body; and then, with many precautions, she was to leave the hospital on the following day, Sept. 29: but that very night she died almost suddenly, having only complained in the evening of a severe pain in the loins." 362.

The brain was very soft. No fluid in the ventricles. The kidneys were larger than usual—of a pinkish-yellow colour, and injected—surface slightly speckled with opake white spots—tunics healthy, but pale. The ovaries were enlarged and round, and darkly injected. The uterus was lined with a thin layer of grumous blood.

We think the appearances in this case were not very satisfactory. There were more evidences of functional disorder than of organic disease in the kidneys.

*Case* 8. An Irishman, aged 50, who had been seven years in the navy, was admitted on the 3d Feb. 1835. He had considered himself well till within the last two years. He attributed his illness to exposure to wet and cold for a whole day. He drank a good deal of spirits on that day. On the next, he found his abdomen rather swollen—penis and scrotum œdematous—pain in the head. He has never since been well. He had been four times salivated, by medical men of " good name and repute."

" The present symptoms are, occasional pain and heaviness of the head, felt principally at night; mouth still under the influence of mercury; cheeks puffed; difficulty of breathing, especially whilst in the horizontal posture; cough, which is most urgent at night; some expectoration; sound of heart unnaturally diffused; palpitation on slightest exertion; abdomen considerably distended, and fluctuation distinct; penis and scrotum, together with both upper and lower extremities, œdematous; pupils dilated, but not insensible to light: vision in both eyes has been imperfect for about eight days, so that he cannot recognize any one at the distance of four or five yards." 364.

We need not detail the treatment. He died on the 23d of the same month. No examination was allowed.

*Case* 9. H. M. aged 43, was admitted, on the 16th Dec. 1835, with general œdema, sallow countenance, and pale lips. He passed a large quantity of urine, highly coagulable. He had much cough and expectoration. He had been 23 years in the marines (a tailor), and lived intemperately; but generally enjoyed good health. A year ago he had acute rheumatism and dropsy; since which he had suffered from cough. On the 27th of the same month, he left the hospital without permission, and three days afterwards he died.

"We learnt that, on leaving the hospital, he walked home, beyond the town; had a single pint of porter; and expressed his intention of resuming his employment next day: but, in the evening he complained of pain in the head, and most intense and excruciating pain in the abdomen, which his sister-in-law said she could compare to nothing but labour-pains, rapidly succeeding each other; and he was at times delirious; he likewise suffered from excessive shivering and coldness; so that he had a large fire made, and sat before it; opening the blanket in which he was enveloped to admit the heat. This state of things continued more or less through the whole of the following day and night; and on the next day, about two o'clock, having for two hours before his death become apparently blind, he died; little more than forty-eight hours having then elapsed from the time when he left the hospital.

*Sectio Cadaveris.* The arachnoid was slightly opaque, particularly along the middle of the sulci of the convolutions; and there was rather more fluid than natural, both beneath it and in the ventricles. The lungs were healthy and crepitant, without adhesion to the pleuræ: the heart large and muscular, but the valves healthy. The abdomen contained a full pint and a half of serum, evidently the result of acute inflammation; it had nearly the consistence of very thin arrow-root or gum-water, opaque, but not puriform. The liver was quite healthy in its structure; but had an unnaturally light and opaque appearance, owing to a thickened condition of its peritoneum. The kidneys were diseased to the utmost degree: they adhered very strongly to their tunics; were lobulated in shape, large, and almost white, with a slight tinge of yellow. When cut into, they were almost cartilaginous in consistence, shewing the same perfectly white colour, with specks of yellow throughout the cortical part; the tubuli presenting a very strong contrast, by their deep red colour. Of these kidneys a very beautiful model was executed in wax by Mr. Town, and is deposited in the Museum." 366.

*Case* 10. This need not detain us long. It is contained in a single page of Dr. B.'s report. The patient, aged 21, was admitted with general anasarca, dingy urine, acid and coagulable—pain in the loins—skin hot and dry. He was confined to bed, and antimonials and Dover's powder administered. Cupped on the loins—and subsequently a seton inserted there. His stomach was peculiarly irritable. In the second week of the fourth month, from this time, he was seized with acute peritonitis, under which he sunk in three days.

The heart was large—lungs gorged—marks of ancient inflammation in the chest. There were marks of extensive peritonitis, and the abdomen contained a quantity of opake fluid. The kidneys were very large, and, though decomposition had advanced, the diseased mottled condition was clearly perceptible.

" Nothing can be more striking than the similarity which is observable in all these cases. I am not aware of any disease in which the character is more completely preserved, or in which the symptoms more clearly mark a specific form of malady. In the first eight cases, the termination, as well as the progress of the disease, bore the most perfect resemblance; and the peculiar train of cerebral symptoms, by which their advanced stages have been attended, have little analogy, when taken as a whole, with the symptoms of any other cerebral affection. The two last cases differ from the rest only in their mode of termination; and I have related them as the two most recent illustrations of a very frequent result of the disease.

Of the insidious nature of this malady, and its fatal tendency, these cases afford a pretty convincing proof: and the fact, that so many of these have come within my own observation in a limited time, would be tolerable evidence of the extreme frequency of the disease. Yet the cases I have now detailed, but more especially the many more which the length of the present communication obliges me to defer, are chiefly such as I have, without any intention of publication, chanced to enter in my note-book, and form but a small portion of those which I have seen: but, in order to obtain a more accurate idea of the actual prevalence of the disease, it is necessary to have recourse to another species of evidence; and accordingly, in the Winter of 1828-9, I instituted a series of experiments, by taking the patients promiscuously, as they lay in the wards, and trying the effects of heat upon the urine of each, and at the same time employing occasionally other re-agents. The whole number I took amounted to 130; out of which no less than eighteen proved to have urine decidedly coagulable by heat; and in twelve more, traces of albumen were found: giving, therefore, an average of at least one in six, if not one in four of the whole number." 368.

In the year 1832, our authors's friends, Dr. Barlow and Mr. Tweedie, made a more extensive range of experiments on nearly 300 individuals; and the result was, that above 1 in 11 were decidedly affected with the disease. In the year 1835, Mr. John Anderson, Dr. Bright's pupil, made a similar experiment on 141 patients, in which the proportion of cases of albuminous urine were above 1 in 6.

" The average of cases affected with the disease under consideration varies much, as might be expected, in different trials; and some explanations, arising from the experiments of Dr. Barlow, of which I have just spoken, will render it highly probable that the average I had taken, amounting to one in six, went beyond the reality: but, with every deduction, it still remains an incontrovertible fact, that the disease in its various stages, from its earliest functional derangement to the confirmed organic malady, is one of the most frequent, as well as of most fatal occurrence; and I think I am fully borne out in the estimate, which I made at the commencement of this paper, that not less than 500 deaths annually occur in this metropolis, from this single disease." 370.

In the treatment of this formidable disease in the early periods of its attacks, Dr. B. looks to the circumstances with which it is most frequently connected, viz. the suppression of perspiration, and the consequent general inflammatory condition—the most important of all others. By the early removal of these, Dr. B. thinks the immediate danger may be guarded against. Early bleeding is, of course, a paramount remedy; but Dr. B. doubts whether, by the most judicious treatment, a liability to relapse can be obliterated, or the morbid tendency overcome.

"With a view to keeping up the action of the skin, I have been very careful in pointing out to those who have not been confined by the disease, the necessity of wearing an inner dress of flannel at all times. I have suggested the propriety of a residence in some more agreeable climate; but I have never had an opportunity of giving a fair trial to this measure; the disease being, unfortunately, most apt to occur in those who are least able to submit to the absence from business, and the expense incident to a residence abroad. In a case which I have related above, I have little doubt that residence in a more northern climate, adopted by the patient without consultation, was instrumental, aided by imprudent exposure, in hastening the fatal result; and I feel confident that, in more than one case now under my care, the bad consequences of the disease are kept at bay by rigid adherence to the use of flannel dresses. Perhaps, to give full effect to a change of climate, some decidedly southern abode should be chosen; and a residence in one of the more healthy of the West India islands, as St. Vincent's, would probably be beneficial.

With regard to the abstraction of blood as a remedial means, it is chiefly in the commencement of the disease that it is decidedly beneficial; and at that time, combined with diaphoretics and strict confinement to bed, general bleeding, freely practised and quickly repeated, is, I believe, most valuable; I have frequently employed it at much later periods, and with temporary advantage. But when we call to mind the constant loss of albuminous matter which the system is sustaining by the kidneys, and the peculiar pallid hue which the patient usually assumes, we shall pause befoie we venture to afford a temporary alleviation, at a still further expense of the more nutritious and stimulating portions of the blood. Yet there is no doubt that, even in the most advanced stages, a strong tendency to inflammatory action, paiticularly of the peritoneum, often displays itself; and, under these circumstances, nothing but active depletion can meet the emergencies of the case. When the head is affected with pain, throbbing, or giddiness, and the senses are becoming less acute, the indication is by no means so obvious; and, although we may believe that a slow inflammatory process has often been going on, yet this is in many cases, greatly to be doubted, as the symptoms, and many of the appearances after death, admit of a ready solution, from the state of debility and anæmia to which the patient is reduced. Cupping from the nape of the neck generally relieves the symptoms for a time; but blisters are less likely to prove injurious; and perhaps a seton would be found advantageous, though of this I have no experience. When the loins are decidedly affected with uneasiness, or when pressure on the kidneys excites pain, I have known the successive daily application of a few leeches attended by the best results, and I have occasionally introduced a seton." 375.

In several cases which we have treated in private practice, the expression of the patient, after every discharge of urine, has been this:—"*I feel as if I discharged so much of my life, with every evacuation of my water.*"

Dr. B. is favourable to puncture of the extremities, when the serous accumulation presses. We have often found it to give great temporary relief.

"The mode which has generally appeared to me most eligible, is the introduction of a needle into the cellular membrane, giving it a turn or two laterally, so as to break down a few of the meshes, and thus prevent the small orifice fiom closing too quickly. Three or four such punctures, made in the calf of the leg, or the thick part of the thigh, will give egress to a large quantity of serum, and will often continue to discharge for three or four days. I once collected in this way, in the course of four hours, above forty-four ounces of serum; and the puncture continued to flow, affording the greatest relief. The operation should not be repeated for some days, or the part should be varied, as the punctures sometimes inflame even after they have appeared to heal." 374.

Dr. B. in accordance with Dr. Barlow and Dr. Osborne, is averse to the exhibition of mercury in cases of albuminous urine. Yet Dr. Pritchard, of Bristol, certainly one of the most talented physicians of our own days, has advocated a moderate use of this remedy. Dr. Bright, however, is inclined to the disuse of this remedy as a general rule. Perhaps he is right. We should never think of giving mercury merely for the disease of the kidney, whether functional or structural; but, when we consider how many other functions are disturbed, besides those of the kidneys and skin—and how useful are mild alterative doses of mercury in restoring or improving these functions, we may well hesitate in proscribing the remedy in question. The judicious practitioner will often find it useful and necessary to obviate collateral or adventitious symptoms, when he finds that he has little power over the paramount disease. He thus mitigates sufferings which he is unable to conquer—he inspires hope and confidence in his patient—and strews the way to the grave with flowers instead of thorns.

"It may, indeed, be fairly urged that, with such a list of fatal results before us, in which various remedies have been used, it is unjust to fix upon mercury the peculiar reproach of failure. I can only answer that, in general, it has appeared to me, that those who, in the confirmed forms of this renal affection, have abstained from mercury, have broken down more slowly under the disease, than those who have taken it to any extent, more particularly if they have persisted till its constitutional effects have completely manifested themselves. I believe, however, that in the very early stages of the acute renal disease, mercury, given in combination with opium and antimony, and associated with bleeding, may be a useful means of reducing the inflammatory action: and I am still by no means inclined to despair of our arriving at the means of administering very minute doses of this powerful remedy, in such combinations as to lead, by long continuance, to some degree of change in the morbid structure which has taken place in all the advanced cases; or to act more decidedly in promoting the absorption of the morbid deposit, if we shall ever arrive at a knowledge of the probable period when the morbid change is in its truly incipient state. I have hoped, in iodine and in the hydriodate of potash, to find remedies adapted to the furtherance of the same object; but I cannot say that, as yet, my experience has borne out these hopes." 377.

Purgatives have produced decidedly good effects in some cases—particularly in reducing the anasarca. The elaterium, under careful management, has been found very beneficial in our author's hands. We are rather surprised at the mention of "grain doses" of this active medicine. Our readers are aware that we have frequently employed it in dropsical affections; but we would hardly dare to give more than the eighth of a grain—generally only the sixteenth part of a grain, for a dose. Dr. B. however, seems to incline to fractional parts of a grain, rather than a grain.

"With regard to diuretics, I have generally wished to abstain from all, except digitalis; and yet I have not unfrequently found myself almost forced to their adoption, when other remedies have failed, in the hope of restoring, for a time, the secretion of the kidney, which has been so greatly reduced as to threaten an entire suppression. I look upon this class of remedies, however, in the light of a necessary evil in such cases; and do not feel authorized in recommending their employment. Very generally, diuretics seem obviously uncalled for, the secretion being already in excess.

Much as may be effected by active remedies in the acute state of the disease; in its chronic and advanced stages, particularly when any change may be supposed to have slowly taken place in the structure of the kidneys, our hopes must rest upon such remedies as produce a more gradual effect, and whose action is therefore not so obvious, and perhaps not so certain, but frequently, I have no hesitation in saying, most salutary. These remedies must vary according to a variety of circumstances, and must be persevered in for almost an indefinite length of time. Amongst these may be mentioned, small doses of carbonate of soda; uva ursi, in its different preparations; small doses of antimonial remedies; and all these, combined with some anodyne, as the conium, or, still better, the compound ipecacuanha powder. The very careful exhibition of the vinum ferri, the tinct. ferri muriatis, or some other chalybeate preparation, has sometimes appeared to do good for a time, but has not generally been admissible for a continuance. Strict attention to the state of the bowels is also indispensable; and becomes the more necessary, because their function is very apt to be interfered with, and their action to become irregular. Sometimes there is a strong tendency to diarrhœa, and occasionally quite an opposite condition exists; some mild combination, calculated to act gently and regularly, should be used; but the exact form will depend very much on our experience in the individual case, as the object should be to produce an action as nearly like the natural as possible, all unnecessary irritation being avoided." 378.

Diet is not to be neglected—the great rule being, to avoid every thing that deranges the stomach. Flannel should be worn next the skin, and all chills and damp avoided.

Dr. Bright has appended a tabular view of the morbid appearances in 100 cases connected with albuminous urine, to which we must refer our readers. In most of these cases, the renal affection was the prominent feature of the disease; and, in all, the granular structure of the kidney was detected after death. The analytical table alluded to discloses a large number of other organic diseases, besides those of the kidney. We are not at liberty, therefore, to assume that the renal disease was the primary one in all cases. Our author, however, is "inclined to believe that the kidney, is the chief promoter of the other derangements." Although the function is generally, it is by no means invariably, interrupted in this disease. "In almost every case, the first impression which brings on the anasarca, is suppression of perspiration." In this terrible disease, the blood undergoes serious changes. It becomes impoverished, and daily robbed of a portion of its most nutrient constituents. From the defective action of the kidneys, too, urea is frequently found in the blood. The principal lesions, however, display themselves in the organs of circulation and respiration, and in the serous membranes. The heart and the lungs, the pleura, the arachnoid, and the peritoneum have, in a large majority of cases, shewn marks of disease. Of all structures, the pleura has been most frequently affected.

"The deviations from health in the heart are well worthy of observation: they have been so frequent, as to shew a most important and intimate connexion with the disease of which we are treating; while, at the same time, there have been twenty-seven cases in which no disease could be detected; and six others, which, from not having been noted, lead to the belief, that no important deviation from the normal state existed. The obvious structural changes in the heart have consisted chiefly of hypertrophy, with or without valvular disease: and what is most striking, out of fifty-two cases of hypertrophy, no valvular disease whatsoever could be detected in thirty-four: but in eleven of these thirty-four, more or less disease existed in the coats of the aorta; still, however, leaving twenty-two without any probable organic cause for the marked hypertrophy generally affecting the left ventricle. This naturally leads us to look for some less local cause, for the unusual efforts to which the heart has been impelled; and the two most ready solutions appear to be, either that the altered quality of the blood affords irregular and unwonted stimulus to the organ immediately; or, that it so affects the minute and capillary circulation, as to render greater action necessary to force the blood through the distant sub-divisions of the vascular system. The valves chiefly affected have been the semilunar valves of the aorta and the mitral; and in three cases, the tricuspid has been somewhat deranged. In three cases, likewise, the disease of the valves has been unattended by any hypertrophy of the heart." 397.

The principal diseases of the lungs have been œdema and bronchitis. The liver and abdominal organs generally appear to escape with wonderful immunity.

"The foregoing table likewise affords an instructive average of the immediate causes of death in this disease. I have been able to trace the circumstances connected with the conclusion of life in seventy cases; and find, that no less than thirty out of these seventy have died of well-marked symptoms of cerebral derangements, noted under the titles of 'apoplexy,' 'coma,' 'convulsion,' and 'epilepsy.' Eight others have died suddenly. In eight cases, the obstructed condition of the lungs has been the immediate cause of death; and in three, the effusion into the chest has hastened the dissolution. Next to head affections, the most prevalent diseases have been inflammatory attacks in the serous membranes; amongst which are five well marked cases of peritonitis, three of pericarditis, one or two of pleuritis. Diarrhœa and other exhausting diseases have carried off several; and in every case, except two or three, the death appears to have been the result, not of casual disease, but of such events as may be said strictly to belong to the condition of the kidney of which we have been treating." 400.

One other circumstance remains to be noted. In 74 cases the age has been recorded. Four only survived the age of 60. Thirteen had passed the 50th year. Twenty-one died between the age of 40 and 50. Sixteen passed the age of 30, and 19 under that age. The larger proportion, therefore, died before they had attained the meridian of life.

We have now given an extended and minute analysis of this highly valuable paper in the "Guy's Hospital Reports"—a work to which we wish every possible success—as well as to its individual contributors. To Dr. Bright the profession is under great obligations. He has not attempted to dazzle us with brilliant theories, nor astonish us by new and successful remedies. By patient observation and careful induction, he is slowly but certainly treading the path to fame and fortune. To such men as Dr, Bright it is the duty—and we will add, the pleasure of the reviewer to do homage, and award praise. To the petulant, the conceited, the shallow-pated author, criticism ought, and, as far as we are concerned, *will* apply the rod of censure, whenever it is judged necessary and proper.

---

P. S. We ought to have observed that there is appended to Dr. Bright's paper, a short notice from Dr. Barlow, of Bath, promising "remarks on certain specimens of urine imitating albuminous urine"—and also a short extract from a recent work of Mr. Rees—which will come in more appropriately on a future occasion.

